# Stroke risks in women with dysmenorrhea by age and stroke subtype

**DOI:** 10.1371/journal.pone.0225221

**Published:** 2019-11-12

**Authors:** Ming-Hung Lin, Chung-Hsin Yeh, Chih-Hsin Mou, Ya-Wen Lin, Pei-Chun Chen, Yin-Yi Chang, Fung-Chang Sung, Jong-Yi Wang

**Affiliations:** 1 Department of Public Health, China Medical University, Taichung, Taiwan; 2 Department of College Pharmacy and Health Care, Tajen University, Pintung, Taiwan; 3 Department of Neurology, Yuan Rung Hospital, Changhua, Taiwan; 4 Department of Nursing, College of Nursing and Health Sciences, Da-Yeh University, Changhua, Taiwan; 5 Department of Nursing, College of Medicine and Nursing, HungKuang University, Taichung, Taiwan; 6 Management Office for Health Data, China Medical University Hospital, Taichung, Taiwan; 7 School of Nursing and Graduate Institute of Nursing, China Medical University, Taichung, Taiwan; 8 Department of Obstetrics and Gynecology, China Medical University Hospital, Taichung, Taiwan; 9 Department of Health Service Administration, China Medical University, Taichung, Taiwan; University of Ioannina School of Medicine, GREECE

## Abstract

**Background:**

Dysmenorrhea and stroke are health problems affecting women worldwide in their day-to-day lives; however, there is limited knowledge of the stroke risk in women with dysmenorrhea, and there have been no studies assessing the specific distribution of stroke subtypes. This case-control study assessed stroke subtypes by age and the role of comorbidities in women with dysmenorrhea.

**Methods and findings:**

Data obtained between 1997 and 2013 from Taiwan’s health insurance database identified 514 stroke cases and 31,201 non-stroke controls in women with dysmenorrhea aged 15–49 years. Proportional distributions of subtypes and odds ratios (ORs) of stroke associated with comorbidities by age and subtype were measured. We found that the stroke risk in dysmenorrheal patients increased with age, and that hypertension was nine-fold more prevalent in the stroke cases than in the controls and was associated with an adjusted OR of 4.53 (95% confidence interval (CI) = 3.46–5.92) for all stroke cases. Moreover, the proportion of hemorrhagic stroke was greater than that of ischemic stroke in younger dysmenorrheal patients between 15–24 years old (50.5% vs. 11.4%), whereas this was reversed in those aged 30–49 years old (16.1% vs. 21.0%). Overall, 25.3% of the stroke cases consisted of transient cerebral ischemia and 31.3% were other acute but ill-defined cerebrovascular diseases, in which the prevalence increased with age for both types of strokes. Hypertension was the comorbidity with the highest OR associated with each subtype stroke; diabetes, hyperlipidemia, arrhythmia, and thyroid disease were also comorbidities that were significantly associated with ill-defined cerebrovascular diseases.

**Conclusions:**

The stroke type varies by age in dysmenorrheal patients, and hypertension is the most important comorbidity associated with all types of stroke; therefore, more attention for stroke prevention must be paid to women with dysmenorrhea, particularly when combined with comorbidities.

## Introduction

Dysmenorrhea is known as painful periods that affect the quality of life of an enormous number of women worldwide [1]. Menstrual cramps can be classified as primary or secondary, with the former manifesting without any gynecological disease and the latter accompanied by a pathological condition in the reproductive organs, such as inflammation, uterine myoma, ovarian cysts, and endometriosis [2,3,4]. Primary dysmenorrhea may occur six months to one year after menarche, whereas secondary dysmenorrhea typically occurs after 25 years of age [2]. The major contributory factors relating to dysmenorrhea have been associated with increased secretion of the hormones vasopressin and oxytocin [4].

Hormonal balance can be altered in women with abnormal menstrual patterns and may mediate the risk of hypertension. Estrogen can play a role in vasodilator function [4,5], while androgens may contribute to the pathogenesis of hypertension [6,7]. Furthermore, women with endometriosis are at an increased risk of hypertension and dysmenorrhea [8,9], and blood pressure may thus be affected in dysmenorrheal women. Worse still, extended use of non-steroidal anti-inflammatory drugs (NSAIDs) are attributable to elevated risks for adverse cardiovascular effects, including bleeding, hypertension, heart attack, and stroke, which are commonly sought by women with dysmenorrhea [10–13]. However, it is not clear whether dysmenorrheal women are at a higher risk of stroke because of elevated blood pressure due to altered hormonal levels and the use of NSAIDs.

The detrimental effects for women with dysmenorrhea are further exacerbated by stroke and other conditions, not only for the individual patients themselves but also for their families. Stroke is the third leading cause of death worldwide and is an important medical condition due to the high incidence of disability and mortality [14]. Despite the alarming potential stroke risk among these women, there is a dearth of clinical observations and empirical data on dysmenorrhea as a health concern.

The type of stroke may vary in women with and without dysmenorrhea. In addition, dysmenorrhea also occurs in teens after menarche. However, no study has ever investigated in detail the types of stroke that occur in younger women with dysmenorrhea compared with older women with dysmenorrhea. In this study, we used the large amount of insurance claim data in Taiwan to conduct a stroke case-control study within dysmenorrheal women to identify the role of selected comorbidities in stroke and to evaluate risks by age and stroke type.

## Research methods

### Data sources

We obtained data from the Longitudinal Health Insurance Database 2000 (LHID2000) of the National Health Research Institutes, which is a subset of insurance claims from one million individuals randomly selected from 23.4 million insured people in Taiwan. More than 99% of the residents of Taiwan have been covered by the health insurance system since 1997 [15]. Data on demographic characteristics, inpatient and outpatient care, medication, and relevant surgical procedure codes and examinations are available for the period from 1996 to 2013. The International Classification of Diseases, 9th Revision, Clinical Modification (ICD-9-CM), was used to code the diseases. To ensure the privacy of the participants, all data were linked using surrogate identification numbers. The use of insurance claims data was approved by the Research Ethics Committee of China Medical University and Hospital (CMUH-104-REC2-115).

### Identification of stroke cases and controls

**Fig 1:**
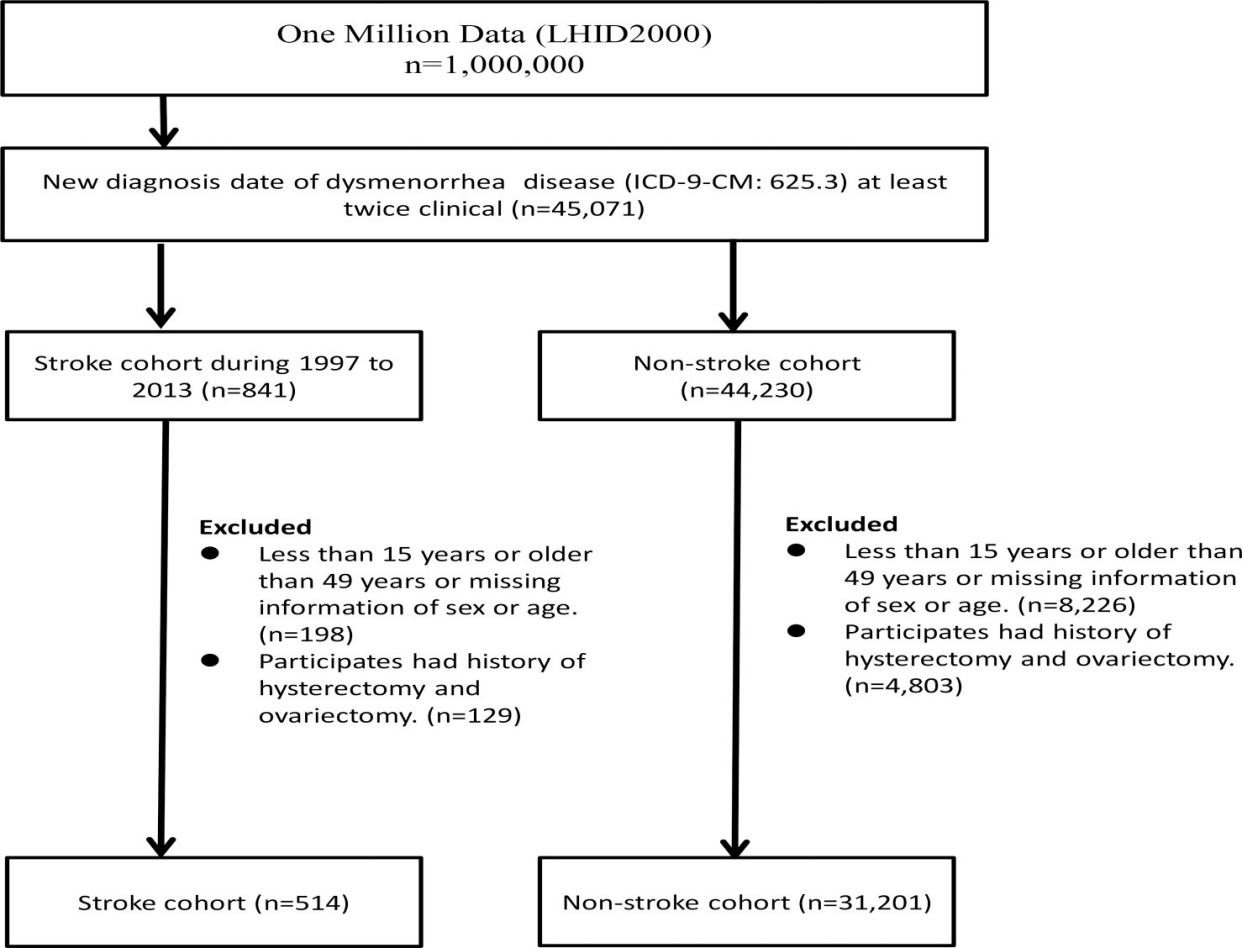
Flowchart identifying stroke cases and controls in women with dysmenorrhea.

From the LHID2000 data set between 1997 and 2013, we identified adolescents and women aged 15–49 years who had sought treatment for dysmenorrhea (ICD-9-CM 625.3) at least twice (Fig 1). From the patients with dysmenorrhea, we identified cases newly diagnosed with stroke (ICD-9-CM 430–437) from 1997 to 2013 as potential stroke cases. The stroke case group was defined after excluding individuals with missing data, those younger than 15 years of age and older than 49 years of age, and those with a history of ovariectomy and/or hysterectomy. All other women diagnosed during the same period who had dysmenorrhea, were aged 15–49 years, were free of stroke, and without an ovariectomy and/or hysterectomy were selected as controls. The demographic data file provided information on birth date, urbanization level of residential area, income, and occupation. We also searched for comorbidities that were potentially linked to stroke risk, including diabetes mellitus (DM) (ICD-9 code: 250), hyperlipidemia (ICD-9 code: 272.x), obesity (ICD-9 code: 278, A183), hypertension (ICD-9 code: 401–405), thyroid disease (ICD-9 code: 420–426), and arrhythmia (ICD-9 code: 427). Medications (cyproterone acetate, ethinyloestradiol, norgestrel, and NSAID) used for dysmenorrhea were also identified.

### Data analysis

Data analysis involved the use of logistic regression analysis as the statistical method. Baseline demographic statuses and comorbidities were compared between the cases and controls. Logistic regression analysis was used to calculate the crude and adjusted odds ratios (cOR and aOR) of stroke and the 95% confidence intervals (CI) to identify whether these variables were also associated with stroke risk. Ages were stratified into subgroups of 15–19, 20–24, 25–29, 30–39, and 40–49 years of age. Urbanization of residential area was stratified into five levels based on population density. We stratified incomes into four levels: <20,000, 20,000–39,999, 40,000–59,999, and >60,000 NTD per month. Occupations included government and school employees, private enterprise employees, self-employed, farmers and other labor workers, and low-income households. Proportional distributions of stroke cases by age and stroke subtype (subarachnoid hemorrhage (SAH, ICD-9 code 430), intracerebral hemorrhage (ICH, ICD-9 code 431), other intracranial hemorrhage (OIH, ICD-9 code 432), ischemic stroke (IS, ICD-9 code 433 and 434), transient cerebral ischemia (TIA, ICD-9 code 435), and other acute and ill-defined cerebrovascular disease (ICD-9 code 436 and 437)) were presented. We further used logistic regression analysis to calculate the ORs of hemorrhagic stroke (SAH, ICH, and OIH), ischemic stroke, TIA, and other ill-defined cerebrovascular disease by age and comorbidity.

## Results

This study included 514 stroke cases and 31,201 non-stroke controls, with mean ages of 34.5 (*SD* = 9.39) and 28.2 (*SD* = 8.38) years, respectively (Table 1). The number of stroke cases and risk of stroke increased with age, with an aOR of 3.95 (95% CI = 2.73–5.72) for those aged 40–49 years, compared to the 15–19 age group. Dysmenorrheal patients with an income of NTD 40,000–59,999 per month were also at an increased risk of stroke.

**Table 1:** Comparison of demographic status between stroke cases and non-stroke controls in dysmenorrheal women (N = 31,715).

Variable	Stroke				Odds ratio (95% CI)	
		No N = 31201		Yes N = 514		
	n	%	n	%	Crude	^a^ Adjusted
Age, years						
15–19	5936	19.0	45	8.75	1.00	1.00
20–24	6892	22.1	60	11.7	1.15(0.78–1.69)	1.13(0.77–1.67)
25–29	6497	20.8	62	12.1	1.26(0.86–1.85)	1.18(0.80–1.74)
30–39	8359	26.8	164	31.9	2.59(1.86–3.61)	2.15(1.52–3.04)^2*^
40–49	3517	11.3	183	35.6	6.86(4.94–9.54)	3.95(2.73–5.72)^3*^
Age mean (SD) ^a^	28.2 (8.38)		34.5 (9.39)			
Urbanization level						
1 (highest)	9342	30.0	159	30.9	1.00	1.00
2	9481	30.4	157	30.5	0.97(0.78–1.22)	0.97(0.77–1.22)
3	5791	18.6	70	13.6	0.71(0.54–0.95)	0.79(0.59–1.05)
4	4038	13.0	82	16.0	1.19(0.91–1.56)	1.28(0.96–1.71)
5 (lowest)	2533	8.12	46	8.95	1.07(0.77–1.49)	1.11(0.75–1.62)
Income per month, NTD						
<20,000	27576	88.4	406	79.0	1.00	1.00
20,000–39,999	2801	8.98	76	14.8	1.84(1.44–2.36)	0.98(0.74–1.28)
40,000–59,999	636	2.04	29	5.64	3.10(2.11–4.55)	1.56(1.03–2.36)^1*^
≥ 60,000	188	0.60	3	0.58	1.08(0.35–3.41)	0.62(0.19–2.02)
Occupation						
Government, school employees	3468	11.1	44	8.56	1.00	1.00
Private enterprise employees	14660	47.0	225	43.8	1.84(1.44–2.36)	0.98(0.74–1.28)
Occupational member	7017	22.5	137	26.7	3.10(2.11–4.55)	1.56(1.03–2.36) ^1*^
Farmers, fishermen	3518	11.3	58	11.3	1.08(0.35–3.41)	0.62(0.19–2.02)
Low-income households and veterans	2526	8.10	50	9.73	1.00	1.00

^a^ Adjusted OR: multiple analysis after adjusting for age, urbanization level, insurance premium, comorbidities, and medication; CI, confidence interval.

1*, *p* <0.05

2*, *P* <0.01

3*p <0.001

Among comorbidities, the patients with hypertension had the strongest relationship with stroke (aOR 4.53, 95% CI = 3.46–5.92) (Table 2). Hyperlipidemia, arrhythmia, and thyroid disease were also significantly associated with a higher risk of stroke. Finally, progestin medications significantly increased the risk of stroke, whereas NSAIDs reduced the risk, however this was not significant.

**Table 2:** Logistic regression analysis measuring the odds ratio of stroke associated with comorbidities and medications in dysmenorrheal women (N = 31,715).

Variable	Stroke				Odds ratio (95% CI)	
	No (N = 31201)		Yes (N = 514)			
	n	%	n	%	Crude	^a^ Adjusted
Diabetes	628	2.01	48	9.34	5.02(3.69–6.82)	1.34(0.93–1.93)
Hypertension	709	2.27	106	20.6	11.1(8.91–14.0)	4.53(3.46–5.92)
Hyperlipidemia	1118	3.58	86	16.7	5.41(4.26–6.87)	1.60(1.19–2.15)
Obesity	288	0.92	15	2.92	3.23(1.91–5.46)	1.21(0.68–2.15)
Arrhythmia	927	2.97	56	10.9	3.99(3.00–5.31)	1.80(1.31–2.46)
Thyroid disease	2077	6.66	80	15.6	2.59(2.03–3.29)	1.56(1.20–2.02)
Medications	15512	49.7	291	56.6	1.32(1.11–1.57)	0.89(0.74–1.07)
Progestin	1460	4.68	38	7.39	1.63(1.16–2.27)	1.19(0.83–1.70)
NSAIDs	14052	45.0	253	49,2	1.22(1.10–1.58)	0.88(0.72–1.06)

^a^ Adjusted OR: Multivariable analysis including age, urbanization level, income, comorbidities, and medications. Progestin medications used included *cyproterone acetate*, *ethinyloestradiol*, *and norgestrel*.

Table 3 shows that the proportional distribution of stroke by subtype in the dysmenorrheal patients varied across age groups. Overall, 25.3% of the stroke cases were hemorrhage events and 18.1% of the cases were ischemic, while 25.3% of the cases were TIA and 31.3% of the cases were ill-defined cerebrovascular disease. Younger dysmenorrheal patients had a higher proportional incidence of hemorrhage events (44.5% in patients who were 15–19 years old and 55.0% in those who were 20–24 years old). The incidence then reduced with age, to 12.6% in patients aged 40–49 years old.

**Table 3:** Distribution of stroke cases by age and stroke subtype in dysmenorrheal women (N = 514).

Age, years	Stroke type													
	SAH		ICH		OIH		IS		TIA		Other		Total (N = 514)	
	n	%	n	%	n	%	n	%	n	%	n	%	n	%
15–19	1	2.22	12	26.7	7	15.6	7	15.6	8	17.8	10	22.2	45	100
20–24	9	15.0	20	33.3	4	6.67	5	8.33	6	10.0	16	26.7	60	100
25–29	5	8.06	14	22.5	2	3.23	8	12.9	15	24.2	18	29.0	62	100
30–39	13	7.93	17	10.4	3	1.83	39	23.8	43	26.2	49	29.9	164	100
40–49	6	3.28	14	7.65	3	1.64	34	18.6	58	31.7	68	37.2	183	100
Total	34	6.61	77	15.0	19	3.70	93	18.1	130	25.3	161	31.3	514	100

SAH: subarachnoid hemorrhage (ICD-9-CM 430); ICH: intracerebral hemorrhage (431); OIH: other intracranial hemorrhage (432); IS: ischemic stroke (433, 434); TIA: transient cerebral ischemia (435); Other: acute but ill-defined cerebrovascular diseases and other ill-defined cerebrovascular diseases (436, 437)

Table 4 details the specific estimated relative risks of stroke subtypes associated with age and comorbidity. The stroke risk increased with age with the exception of the hemorrhagic stroke subtype, which was higher in those aged 25–29 years. The aOR of ischemic stroke increased to 4.60 (95% CI = 1.87–11.3) for the 40-49-year-old dysmenorrheal patients, while the aORs for TIA and ill-defined cerebrovascular disease were both greater than 6.0. Hypertension was the comorbidity with the strongest risk associated with each subtype stroke. Obesity was only associated with hemorrhagic stroke, whereas ill-defined cerebrovascular disease was associated with diabetes, hyperlipidemia, arrhythmia, and thyroid disease. Medications were also associated with a lower risk of stroke.

**Table 4:** Adjusted odds ratio of stroke subtype associated with age, comorbidity, and medication in dysmenorrheal women (N = 31,715).

Variable	Non-stroke N = 31201		Hemorrhagic N = 130			Ischemic N = 93		
	n	%	n	%	Adjusted odds ratio (95% CI)	n	%	Adjusted odds ratio (95% CI)
Age, years								
15–19	5936	19.0	20	15.4	1.00	7	7.53	1.00
20–24	6892	22.1	33	25.4	1.48 (0.85–2.60)	5	5.38	0.61 (0.19–1.92)
25–29	6497	20.8	21	16.2	0.96 (0.51–1.80)	8	8.60	0.99 (0.35–2.76)
30–39	8359	26.8	33	25.4	1.02 (0.56–1.85)	39	41.9	3.22 (1.40–7.42)
40–49	3517	11.3	23	17.7	1.16 (0.56–2.38)	34	36.6	4.60 (1.87–11.3)
Diabetes	628	2.01	8	6.15	1.62 (0.70–3.73)	8	8.60	1.14 (0.50–2.61)
Hypertension	709	2.27	17	13.1	5.49 (2.95–10.2)	23	24.7	5.84 (3.34–10.2)
Hyperlipidemia	1118	3.58	10	7.69	0.96 (0.44–2.07)	17	18.3	1.75 (0.93–3.30)
Obesity	288	0.92	5	3.85	2.78 (1.06–7.33)	2	2.15	0.77 (0.18–3.37)
Arrhythmia	927	2.97	5	3.85	0.90 (0.35–2.28)	8	8.60	1.26 (0.58–2.74)
Thyroid disease	2077	6.66	9	6.92	0.90 (0.45–1.81)	12	12.9	1.20 (0.64–2.27)
Medication	15512	49.7	58	44.6	0.76 (0.52–1.09)	54	58.1	0.92 (0.60–1.42)
	Non-stroke N = 31201		TIA N = 130			Others ^b^ N = 161		
Age, years								
15–19	5936	19.0	8	6.15	1.00	10	6.21	1.00
20–24	6892	22.1	6	4.62	0.59 (0.20–1.70)	16	9.94	1.38 (0.63–3.05)
25–29	6497	20.8	15	11.5	1.43 (0.60–3.41)	18	11.2	1.57 (0.72–3.43)
30–39	8359	26.8	43	33.1	2.85 (1.31–6.20)	49	30.4	2.97 (1.47–6.00)
40–49	3517	11.3	58	44.6	6.25 (2.79–14.0)	68	42.2	6.82 (3.30–14.1)
Diabetes	628	2.01	11	8.46	0.96 (0.47–1.95)	21	13.0	1.95 (1.23–3.12)
Hypertension	709	2.27	31	23.9	4.66 (2.87–7.55)	35	21.7	3.47 (2.19–5.48)
Hyperlipidemia	1118	3.58	23	17.7	1.49 (0.86–2.57)	36	22.4	1.95 (1.23–3.12)
Obesity	288	0.92	3	2.31	0.88 (0.26–2.96)	5	3.11	1.01 (0.39–2.63)
Arrhythmia	927	2.97	19	14.6	2.23 (1.03–3.81)	24	14.9	2.12 (1.30–3.46)
Thyroid disease	2077	6.66	20	15.4	1.29 (0.78–2.14)	39	24.2	2.51 (1.70–3.73)
Medication	15512	49.7	90	69.2	1.40 (0.94–2.07)	89	55.3	0.70 (0.50–0.98)

Note: the sum of all subarachnoid, intracerebral, and other intracranial hemorrhage patients was used when the hemorrhagic group was calculated.

^a^ Adjusted OR: Multivariable analysis including age, urbanization level, income, occupation, comorbidities, and medications. Medications used include *cyproterone acetate*, *ethinyloestradiol*, *norgestrel*, *and NSAIDs*.

^b^ Other: Acute and other ill-defined cerebrovascular diseases

## Discussion

Previous research on stroke has attributed an increased stroke risk to hypertension and hormonal imbalances brought on by diabetes mellitus or thyroid disease, among others [16–18]. The present study assessed the subtypes of stroke in women with dysmenorrhea and comorbidities, which have been thought to increase the likelihood of stroke. There were fewer ischemic strokes compared to other subtypes of stroke but more other acute and ill-defined cerebrovascular diseases than subarachnoid, intracerebral, and other intracranial hemorrhages. In particular, women with both dysmenorrhea and hypertension were nearly nine-fold more likely than women in general with hypertension to suffer a stroke, with an aOR of 4.53; this indicated that dysmenorrhea and hypertension can interact and strongly increase the risk of stroke. The risk of stroke also increased with age.

It should be noted that unlike previous studies, which have suggested that the prevalence of dysmenorrhea is high among women in their 10s and 20s [19,20], this study found that the condition was more prevalent among controls aged 20–39 years (69.7%) and among cases aged 30–49 years (67.5%). The disparity could be linked to the fact that this study did not separate the type of dysmenorrhea that was investigated–it is known that secondary dysmenorrhea is more prevalent among elderly women (>35 years). Moreover, the types of stroke vary among different age groups of women with dysmenorrhea. Therefore, the novel findings in this study were that the types of stroke differ among age groups as well as between women with dysmenorrhea and women in general. The overall portion of hemorrhagic stroke was greater than that of ischemic stroke (25.3 vs. 18.1%), and the gap was lower between dysmenorrheal patients aged 15–24 and those aged 40–49 (50.5 vs. 12.6%). On the other hand, the ratio of hemorrhagic stroke was lower than that of ischemic stroke in the 30-49-year-old group (a ratio of 1:1.3, or 16.1% vs 21.0%), which was different from the ratios measured for the general population in other studies. An earlier study in Taiwan found that the ratios were 1:2.6 (19.3% vs. 49.6%) in general women aged 20 and older [21] and 5.3:1 (58.3% vs. 11.1%) in girls aged 10–19, the latter of which was somewhat similar to the dysmenorrheal patients aged 15–24 in the present study. In addition, an earlier US study reported a higher incidence of ischemic stroke than hemorrhagic stroke in children (7.8 vs. 2.9 per 100,000) [22]. Children in Hong Kong and Dijon, France also have higher incidences of ischemic stroke than hemorrhagic stroke [23,24]. The results of our study were inconsistent compared with previous studies, possibly due to limitations such as obtaining data only from the Taiwan health insurance database.

It is also important to note that 25.3% of the stroke cases in the dysmenorrheal women were TIA with minor symptoms. On the other hand, 31.3% of the cases had acute and other ill-defined symptoms, in which the incidence also increased with age (37.2% for those aged 40–49).

The findings in this study indicated diabetes, hypertension, hyperlipidemia, arrhythmia, and thyroid disease as risk factors for acute and other ill-defined strokes among women with dysmenorrhea, corroborating the findings by a number of previous researchers [25]. It should be noted, however, that this study did not explain the mechanisms through which each of the comorbidities increases the risk of stroke among women with dysmenorrhea.

Women suffering from menstrual cramps are generally prescribed medications, which include NSAIDs and progestin in Taiwan. Our study did not observe progestin and NSAIDs as factors associated with the overall stroke risk. Similarly, a recent study identified no significant association between the risk of stroke in women and the use of contraceptives containing estrogen [26,27]. The use of NSAIDs in the management of dysmenorrhea can interfere with rhythmic contractions of the heart and result in the development of myocardial infarction [28–30]. The disrupted heart functioning can then lead to alterations in blood pressure that have been thought to result in stroke; however, the evidence for a link between NSAIDs and stroke events still remains unclear. It was interesting to note that our study found that medications may reduce acute and other ill-defined cerebrovascular disease by 30%. We also noted that 91% of the dysmenorrheal patients were NSAIDs users.

The findings of this study have major clinical implications in the management of dysmenorrhea and the prevention of stroke. Health practitioners should closely monitor women of all age groups with dysmenorrhea for possible signs of stroke. The risk of stroke among women with dysmenorrhea could be reduced by adopting lifestyle practices and health care plans, particularly for women with comorbidities.

The use of a large amount of population data enabled us to evaluate the risk of stroke subtype by age and comorbidity in women with dysmenorrhea. However, information on detailed comorbidity conditions was unavailable, and thus we were unable to evaluate the stroke risk by levels of blood pressure and other comorbidities. Information on secretion of the hormones vasopressin and oxytocin as well as higher levels of prostaglandin F2α was also unavailable, preventing the evaluation of stroke risk and the relationships with stroke subtypes.

## Limitations

The major limitation for this work was the fact that the data were obtained only from the Taiwan health insurance database, which limited the extent of our findings. Furthermore, some people in Taiwan lack health insurance coverage, and therefore some women who fit our criteria might not have been captured in the insurance database. Therefore, analysis using other databases could lead to findings that differ from the findings in this study. Finally, as our study only captured women with dysmenorrhea aged 15 to 49 years from 1997–2013, it is likely that a higher number of women suffering from dysmenorrhea did not fall within the age brackets that we analyzed.

## Conclusion

In this study, we identified various factors that increase the risk of stroke among women suffering from dysmenorrhea. Women with the comorbidities of hypertension, hyperlipidemia, arrhythmia, and thyroid disease have a higher risk of stroke. This study also revealed that younger women with dysmenorrhea are at a higher risk for hemorrhagic stroke, whereas older ones are at a higher risk for acute and other ill-defined strokes. This study supported previous studies that recommend reducing the risk of stroke through the prevention of diabetes, hypertension, hyperlipidemia, arrhythmia, and thyroid disease. Furthermore, specific to women with dysmenorrhea, this study recommended that methods which ensure the prevention of hypertension, hyperlipidemia, arrhythmia, and thyroid disease be adopted so as to reduce the risk of stroke. Hemorrhagic stroke prevention for younger dysmenorrheal women also merits greater attention. Finally, in older women, the prevalence of ischemic strokes and TIAs was greater than that of hemorrhagic strokes.

## Abbreviations

ICD-9-CM: International Classification of Diseases, 9th Revision, Clinical Modification
ICH: intracerebral hemorrhage
IS: ischemic stroke
LHID2000: Longitudinal Health Insurance Database 2000
NSAIDs: non-steroidal anti-inflammatory drugs
OIH: other intracranial hemorrhage
OR (95% CI): odds ratio and related 95% confidence interval
SAH: subarachnoid hemorrhage
TIA: transient cerebral ischemia
